# Traumatic Transection of Descending Thoracic Aorta Presenting as Pseudo- Coarctation

**Published:** 2018-10

**Authors:** Shadi Shafaghi, Neda Behzadnia, Babak Sharif-Kashani, Farah Naghashzadeh, Zargham Hossein Ahmadi

**Affiliations:** 1 Lung Transplantation Research Center, National Research Institute of Tuberculosis and Lung Diseases (NRITLD), Shahid Beheshti University of Medical Sciences, Tehran, Iran,; 2 Tobacco Prevention and Control Research Center, NRITLD, Shahid Beheshti University of Medical Sciences, Tehran, Iran

**Keywords:** Hypertension, Transection of aorta, Traumatic aortic injury

## Abstract

**Background:** Thoracic Aortic Injury (TAI) due to penetrating or blunt chest trauma is a critical life-threatening aortic injury. Its diagnosis and treatment always is challenging.

An 18-year-old male was admitted due to blunt chest trauma after a high-impact road traffic collision. According to presenting dyspnea, an emergency chest-x-ray revealed left hemothorax for which chest tube was inserted. Hemodynamic monitoring demonstrated uncontrolled hypertension with systolic blood pressure of 200–220 *mmHg* despite multiple anti-hypertensive drugs. Trans-Thoracic Echocardiography (TTE) revealed abnormal Doppler flow pattern in proximal descending thoracic aorta suggestive for probable coarctation of aorta. Chest CT scan revealed pseudoaneurysm of the descending thoracic aorta just below the isthmus. Due to uncontrolled hypertension, persistent hemothorax and probable aortic pseudoaneurysm presenting as aortic luminal narrowing, surgical resection of the aneurysm was planned.

The postoperative course was uneventful and blood pressure normalized without any drugs. Patient is normotensive after 8 years follow up.

## INTRODUCTION

Thoracic Aortic Injury (TAI) due to penetrating or blunt (more common) chest trauma is the most frequent type of traumatic aortic injury and is a catastrophic and also final scenario in most circumstances (1). Blunt Traumatic Thoracic Aortic Injury (BTTAI) is a devastating clinical scenario associated with disappointing prognosis if undetected, as shown by studies reporting early mortality in more than 75% of cases (2–4). According to data from two separate studies in 1985, TAI was linked with 16 to 23% of all motor vehicle fatalities (4, 5). Also transection of the aorta is seen with a variety of non-vehicular accidents that result in sudden deceleration and stress at aortic isthmus and transition zone of aortic arch to proximal descending thoracic aorta. A major vascular cause of hemothorax is rupture of thoracic aortic pseudoaneurysm or aortic dissection (6). Its diagnosis and treatment always is challenging. Nearly 80% of patients with TAI are lost immediately at the scene of the accidents. Coexisting devastating injuries are frequent and clinical picture is often nonspecific and obscured. Clinical presentation may include chest or mid-scapular back pain, signs of external chest trauma or hemodynamically shock state. Clinical suspicion is usually based on mechanism and serious nature of the trauma. The diagnosis ultimately relies on appropriate imaging studies. The prognosis of blunt traumatic TAI is mostly dependent on the severity of the non-aortic injuries and delayed aortic surgical repair is even advisable as compared with immediate repair to get time for hemodynamic stability and urgent care of otherwise critical non-aortic traumatic injuries (4, 5).

As aortic dissection may be associated with a hypertensive crisis, the patients must be ICU admitted and dedicated control of blood pressure is very important to reduce the progressive process of dissection.

Once the clinical suspicion of acute aortic dissection is suggested, intravenous (IV) antihypertensive regimen should be started as soon as possible in emergency department in all non-hypotensive patients and continued in the intensive care unit or the operation room. Systemic blood pressures, urine output, consciousness, and focal neurologic signs should be regularly checked for any derangement owing to complications.

Surgical techniques can be open surgery with a clamp-and-sew technique, surgery with the use of a vascular graft, and using heparin-less partial cardiopulmonary bypass. Recently new endovascular techniques have been largely introduced as a therapeutic choice in traumatic and non-traumatic aortic injury in descending and abdominal aorta.

## CASE SUMMARIES

An 18-year-old man with shortness of breath and left sided bloody pleural effusion was admitted in this center. The patient did not have any underlying disease. The patient was pale and bilateral lower extremity pulses were not palpable. There was history of blunt chest trauma after an acceleration- deceleration traffic collision 12 days ago. After resuscitation and stabilization of the patient, because of dyspnea and hemothorax, an emergency chest-x-ray showed left hemothorax and chest tube was inserted. Patient referred to tertiary lung disease center due to persistent dyspnea and admitted to ICU because of low O2 saturation. Hemodynamic monitoring in ICU demonstrated severe refractory uncontrolled hypertension with systolic blood pressure of 200–220 *mmHg* despite multiple anti-hypertensive drugs. Another CT scan revealed pseudoaneurysm of the descending thoracic aorta.

**Figure 1.**
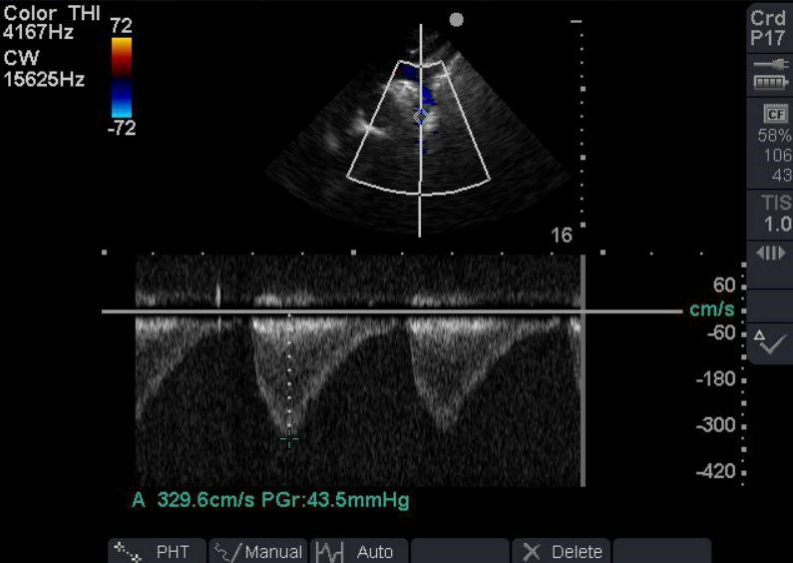
Abnormal continuous Doppler flow pattern in proximal descending aorta from suprasternal notch consistent with coarctation physiology.

**Figure 2.**
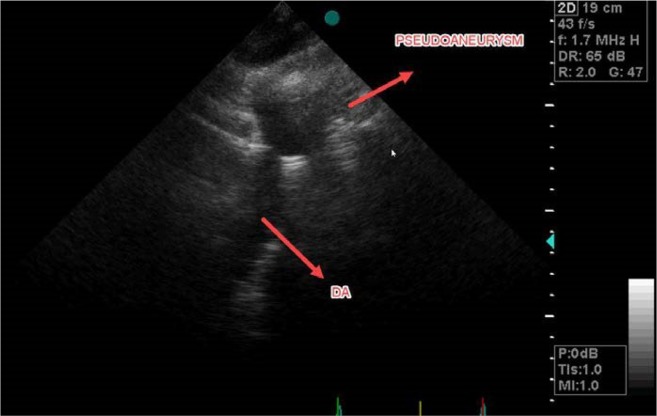
Suprasternal echo window showed pseudoaneurysm of proximal descending aorta and aortic luminal narrowing in post- traumatic patient.

**Figure 3.**
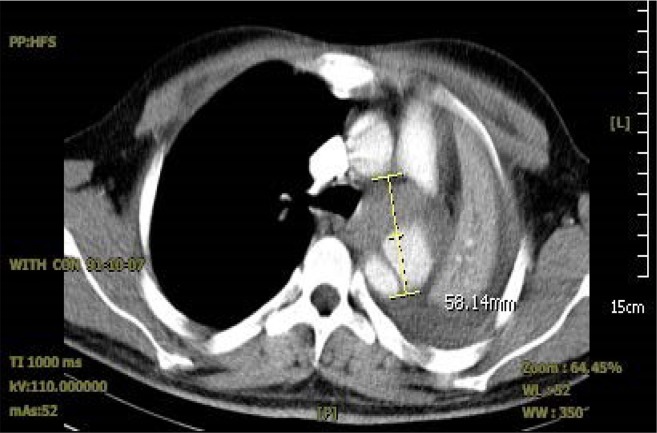
Contrast-enhanced Spiral Chest showing soft tissue mass in aortopulmnary (AP) window measure up to 58×42×45 mm which was partially contrast filled with continuity with descending aorta and compressing left main bronchus. Findings are mostly in favor of aortic pseudo aneurysm. Left lung is totally collapsed with pleural effusion ipsilateraly.

Bedside Trans-Thoracic Echocardiography (TTE) from suprasternal notch revealed abnormal continuous Doppler flow pattern in proximal descending thoracic aorta suggestive for probable coarctation of aorta (Figure 1) and aortic luminal narrowing just after the isthmus (Figure 2). CT angiography illustrated a hematoma around the descending aorta after just below the isthmus suggestive for pseudoaneurysm (Figure 3) as cause of pseudocoarctation presentation. Due to uncontrolled hypertension and persistent hemothorax and probable aortic isthmus pseudoaneurysm presenting as aortic luminal narrowing, surgical resection of the aneurysm was planned.

The patient underwent left sided thoracotomy and a transection of aorta in the posterior surface of aorta just after the isthmus was found which resulted in pseudoaneurysm formation. After excision, the aorta was repaired with a 16 mm Gore-Tex interposing graft (GORE-TEX® Vascular Grafts, W. L. Gore & Associates, Inc., Flagstaff, AZ, U.S.A.).

The postoperative course was uneventful and blood pressure normalized without any drugs and the patient had full recovery on the 7^th^ post-operative day. Follow up imaging with Magnetic Resonance Imaging (MRI) revealed normal anatomy of the aorta without luminal narrowing two weeks after operation. No complication occurred and patient had normal blood pressure after 8 years follow up.

## DISCUSSION

Traumatic damages to aorta are accomplished with wide range of aortic damages including minimal aortic injury, aortic laceration or transverse aortic tearing. Aortic transection is also known as aortic rupture, aortic pseudoaneurysm and aortic intramural hematoma.

Supine chest-x-ray is the initial screening test in the traumatic patient. A large autopsy survey reported that 97% of victims of aortic injury had additional traumatic damages in other regions. Indirect signs suggestive for aortic injury are mediastinal hematoma and widening or concomitant multiple rib fractures. Other imaging tests such as contrast enhanced-CT scan, Magnetic Resonance Angiogram (MRA), or aortic angiogram may be used to determine if aortic tear is present. Another noninvasive diagnostic test that may be used is bedside echocardiography that had been conducted for this patient and demonstrated TAI.

Regarding the mechanism of refractory hypertension in this case, aortic luminal narrowing or kinking just after aortic injury site in isthmus with a pseudocoarctation physiology presenting as continuous Doppler flow pattern in descending aorta was completely normalized after successful surgical aortic repair.

There are different guidelines for medical treatment of hypertension in these patients (7). Over the last decade, new evolving techniques in treatment of aortic dissection and thoracic aortic aneurysms have been introduced including endovascular aortic replacement with excellent results. Therapeutic algorithm include medical, endovascular and surgical interventions (8). If patient is clinically stable, careful management of traumatic aortic injury is associated with satisfactory improvement in survival with no significant difference between surgical and endovascular repair. However, regarding the less invasiveness of endovascular repair, makes it suitable choice in unstable cases or patients with comorbidity and high surgical risk (9). Conventional surgical techniques still are favorable as an alternative to endovascular procedures if anatomy is not ideal or there is need for surgical revascularization of the left subclavian artery, carotid artery, or both (10).

The striking aspects of the case were new-onset refractory hypertension in previously normotensive young man and echocardiographic findings mimicking aortic coarctation as result of traumatic transection of aorta and amenability of lesion to surgery and complete correction of hypertension after surgical repair.

## CONCLUSION

Blunt traumatic TAI is a deadly catastrophe with disappointing survival rates, as confirmed by previous studies and immediate death rate of more than 75% (11, 12). In acceleration-deceleration traumatic injuries with uncontrolled hypertension and aortic pseudocoarctation symptom especially in previously normotensive patients we should consider aortic transection in order to not miss the patient. Echocardiographic modalities are noninvasive and most available imaging techniques that can help in better management of these cases.
